# Effectiveness of a behavioural intervention delivered by text messages (safetxt) on sexually transmitted reinfections in people aged 16-24 years: randomised controlled trial

**DOI:** 10.1136/bmj.o2421

**Published:** 2022-10-07

In this paper by Free and colleagues (*BMJ* 2022;378:e070351, doi:10.1136/bmj-2022-070351, published 28 September 2022), the first two points of the what this study adds in the box should read: The safetxt intervention using a mobile phone and targeting safer sex behaviours did not reduce the incidence of chlamydia or gonorrhoea at one year; more infections occurred in the intervention group. Safetex increased some self-reported measures of sexual health, such as self-efficacy in condom use and condom use in itself. 

